# Effect of the Abutment Rigidity on the Wear Resistance of a Lithium Disilicate Glass Ceramic: An In Vitro Study

**DOI:** 10.3390/jfb14080395

**Published:** 2023-07-25

**Authors:** Przemysław Kosewski, Francesco De Angelis, Edoardo Sorrentino, Agnieszka Mielczarek, Matteo Buonvivere, Camillo D’Arcangelo

**Affiliations:** 1 Private Practice, 01650 Warsaw, Poland; przemekkosewski@gmail.com; 2 Unit of Restorative Dentistry and Endodontics, Department of Medical, Oral and Biotechnological Science, School of Dentistry, “G. D’Annunzio” University of Chieti, 66100 Chieti, Italy; m.buonvivere@gmail.com (M.B.); cdarcang@unich.it (C.D.); 3 Department of Innovative Technologies in Medicine & Dentistry, “G. D’Annunzio” University of Chieti, 66100 Chieti, Italy; sorrentinoed@libero.it; 4 Department of Conservative Dentistry, Medical University of Warsaw, 02091 Warsaw, Poland; agnieszka.mielczarek@wum.edu.pl

**Keywords:** dental materials, dental implants, ceramics, lithia disilicate, tooth wear, dental abutments, 3D printing, periodontium, periodontal ligament

## Abstract

Lithium disilicate (LDS) glass ceramics are among the most common biomaterials in conservative dentistry and prosthodontics, and their wear behavior is of paramount clinical interest. An innovative in vitro model is presented, which employs CAD/CAM technology to simulate the periodontal ligament and alveolar bone. The model aims to evaluate the effect of the abutment rigidity on the wear resistance of the LDS glass ceramic. Two experimental groups (LDS restorations supported by dental implants, named LDS-on-Implant, or by hybrid ceramic tooth replicas with artificial periodontal ligament, named LDS-on-Tooth-Replica) and a control group (LDS-Cylinders) were compared. Fifteen samples (*n* = 15) were fabricated for each group and subjected to testing, with LDS antagonistic cusps opposing them over 120,000 cycles using a dual axis chewing simulator. Wear resistance was analyzed by measuring the vertical wear depth (mm) and the volume loss (mm^3^) on each LDS sample, as well as the linear antagonist wear (mm) on LDS cusps. Mean values were calculated for LDS-Cylinders (0.186 mm, 0.322 mm^3^, 0.220 mm, respectively), LDS-on-Implant (0.128 mm, 0.166 mm^3^, 0.199 mm, respectively), and LDS-on-Tooth-Replica (0.098 mm, 0.107 mm^3^, 0.172 mm, respectively) and compared using one-way-ANOVA and Tukey’s tests. The level of significance was set at 0.05 in all tests. Wear facets were inspected under a scanning electron microscope. Data analysis revealed that abutment rigidity was able to significantly affect the wear pattern of LDS, which seems to be more intense on rigid implant-abutment supports compared to resilient teeth replicas with artificial periodontal ligament.

## 1. Introduction

Natural occlusion (i.e., the way in which the teeth of the upper and lower jaw contact each other) is a state of dynamic equilibrium and the wear of dental tissues is its inherent component [1]. Studies show that occlusion is self-adjusting through the process of wear and continuous tooth migration [2]. Under physiological conditions, tooth enamel wears off at an average rate of 30–35 µm per year [3]. Within certain limits, teeth wear is therefore essential for masticatory efficiency. Predisposing factors, however, are able to increase this physiological wear rate and gradually lead to an occlusal vertical dimension loss jeopardizing not only dental aesthetics, but the stomatognathic system functions as well [4,5,6,7,8,9,10]. Given the subtle boundary between physiological and pathological wear rate, accurate diagnosis is essential. While in the past this relied solely on the clinician’s experience, nowadays it is possible to take advantage of intraoral scanners to perform 3D monitoring of the patients, thereby obtaining objective measurements (such as size and depth of the wear facets) of the patient’s tooth wear over time, in order to determine whether treatment is necessary or not [11,12]. In cases of pathological wear, a clinical intervention with a minimally invasive approach becomes mandatory [13,14,15]. Modern restorative materials with favorable wear behavior, such as lithium disilicate (LDS) glass ceramic (luted with contemporary cements [16,17,18]), make it possible to work in an “additive way”, reestablishing a proper occlusal vertical dimension without sacrificing sound tooth structure [19,20,21]. The periodontal ligament (PDL) is a layer of connective tissue that covers the root cement and forms the tooth’s suspension apparatus. Inside the periodontium there is a dense network of blood vessels and fluid. Not only the connective tissue ligaments but also the fluid pressure in the periodontium and the blood pressure in the blood vessels are responsible for the mechanical properties of the periodontium. The complex functioning of the periodontium and its viscoelastic properties are responsible for enabling the dissipation of energy and absorption of forces exerted on the teeth [22]. Dental implants used to restore missing teeth do not have periodontal ligament nor mechanoreceptors and are not able to move in the bone, which means that the natural mechanisms of adapting to occlusion do not function in their case [23]. Crowns on implants made of ceramic materials or zirconium oxide are additionally characterized by greater hardness and a lower modulus of elasticity [24] as well as lower ability to wear than natural human enamel [25,26].

Dental implants used to restore missing teeth offer a highly predictable solution: meta-analyses show an average of 94.6% implant survival over an average follow-up of 13.4 years [27]. Nevertheless, complications of both biological and mechanical nature are relatively common. It is estimated that on average 24% of implants will develop peri-implantitis [28], whereas the incidence of mechanical failure is estimated to be 5.6% to 7.7% [29]. There are reports that overloading on implants may lead to an increased risk of failure in implant prosthetic treatment, contributing not only to mechanical complications, but also exacerbating the course of biological complications [30,31].

Due to the rigid anchorage of implants in the bone, they are subjected to a different type of load than natural teeth [32]. According to available research, the perception of pressure on implants is 4 to 20 times less sensitive than on natural teeth [33]. An experimental animal study by Cheng et al. comparing the biomechanics of teeth with natural dentition showed that tooth displacement under pressure is many times greater than with implants [22]. This study also showed that the energy dissipation capacity of the periodontium is multiple times greater than that of implants and, with a load of 300 g, a natural tooth was able to dissipate 50 times more energy [22]. The viscoelastic properties of the periodontium are able to absorb energy and transfer it to the surrounding bone, dissipating chewing forces. Implants, on the other hand, have a limited ability to dissipate energy [22]. Data shows that implant-supported prosthetic restorations are more likely to suffer from technical complications compared to crowns on natural teeth. Chippings or fractures of ceramic restorations are more common on implants than on natural teeth (8.8% vs. 2.9% over 5 years of observation) [34]. The restorative material choice, already crucial on natural teeth, becomes even more important in a system with reduced abutment mobility, such as the implant-supported crown. In these terms, LDS restorations, with their lower modulus of elasticity, could be a valid alternative to zirconia [35]. This is so true that LDS crowns are being increasingly used for single-unit implant-supported restorations [36,37,38,39,40,41].

Wear of materials inside the oral cavity is a process influenced by many biological, chemical and physical factors. Due to technological limitations and the presence of many factors affecting the behavior of prosthetic materials in the oral cavity, currently available clinical studies on the abrasion of dental materials or tissues are insufficiently precise [38,42,43,44]. Thus, in vitro tests are currently employed as an important tool for assessing the basic wear behavior of dental materials [45] and their effect on antagonistic materials. Several papers investigated the in vitro wear resistance of restorative materials opposing either human enamel antagonists or dedicated artificial abraders [25,46], underlining the effect of different antagonist materials on the wear rate of tested specimens. Furthermore, few studies analyzed the wear behavior of dental materials opposing themselves, which seems particularly interesting for simulating the clinical scenario of restored occlusal surfaces occluding in a full-mouth rehabilitation [26,47].

Although the complexity of tooth biomechanics and the variability of the oral cavity environment cannot be completely simulated, various methods of restoring the periodontal ligament have been described in the literature in order to take into account the resilience of PDL in mechanical tests [48] and bring the in vitro measurements closer to the clinical situation. The most commonly used approach is to create a layer of elastic material around the root of the tooth using silicone or polyether materials [49]. Previously described methods involved the process of manually soaking the tooth root in wax and then replacing it with a polyether material [50] or manually applying several layers of rubber on the tooth root [51]. Some authors, however, postulated that the production of periodontal spaces from wax could result in poor control of the thickness of the material simulating periodontium, and thus affect the mechanical properties of the models [51,52]. Since a standardized approach on the matter is still lacking, a large variety of methodologies for producing PDL simulations can still be observed in the available literature [48]. Currently, there are no conclusive data in the literature about the effect of different abutment rigidity on the wear of prosthetic materials. One pilot study by Rosentritt et al. described a quadrant model of the jaws including teeth with simulated periodontium and implants [38]. Models were subjected to wear analysis and the wear of the natural enamel on antagonistic teeth was calculated, but no data were obtained regarding wear depth of restorative materials supported by teeth or implants [38].

On the above basis, the aim of the present study was to evaluate the influence of the abutment rigidity on the wear resistance of lithium disilicate (LDS) restorations (supported by either rigid titanium dental implants or elastic hybrid ceramic tooth replicas) using an in vitro model simulating alveolar bone and periodontal ligament. The null hypothesis to be tested was that the abutment rigidity is not able to influence the wear resistance of lithium disilicate restorations.

## 2. Materials and Methods

The present study aimed at comparing two experimental groups (LDS-on-Implant and LDS-on-Tooth-Replica) and a control group (LDS-Cylinders).

The components of the experimental model were computer-designed using Solidworks 2016 SP 5.0 (Dassault Systémes SolidWorks Corporation, Vélizy-Villacoublay, France). The experimental models were developed and validated with the use of Periotest Classic device (Medizintechnik Gulden, Modautal, Germany)—a device used for measurement of mobility of natural teeth and implants, which uses percussion on the surface of the crown to measure the damping capacity, which is expressed as PTV (Periotest Value). PTV ranges from −8 to +50, where a lower PTV is correlated with lower mobility, and higher values with higher mobility of subject tested.

### 2.1. Sample Size Estimation

Sample size was estimated with Statistica 13.3 (StatSoft GmbH, Hamburg, Germany). Independent Sample t-Test was used. The base value for material wear of lithium disilicate and standard deviation of measurements was adopted from research by D’Arcangelo et al. [47]. The anticipated difference of 20% was used for calculations on the basis of a preliminary study by Rosentritt et al. [38] in which the depth of wear traces on prosthetic restorations opposing natural enamel and supported by resin teeth or implants was evaluated (516.8 ± 97.4 and 636.8 ± 187.1 µm, respectively). With statistical power of 0.8 and α = 0.05, the required number of samples per group was estimated at 14.

### 2.2. Sample Fabrication

**Figure 1:**
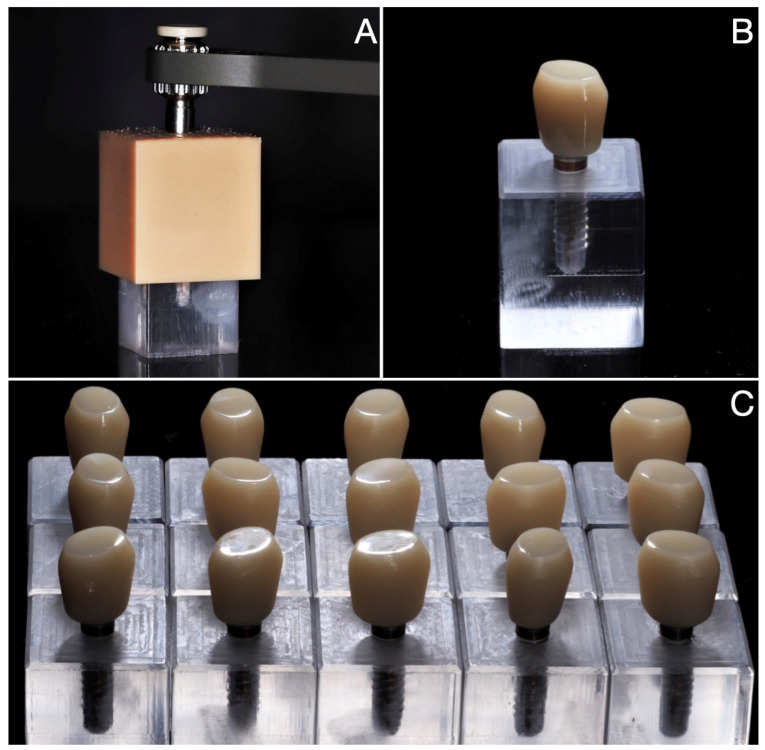
LDS-on-Implant samples. Cylindrical dental implants inserted parallel to the long axis of the implant bed in the blocks with the use of 3D printed guide (**A**). After placing the prosthetic abutments, monolithic lithium disilicate single crowns with a flat occlusal surface were adhesively luted to the implant-abutment complex (**B**). A total of 15 samples was fabricated (**C**).

For the LDS-on-Implant group, fifteen PMMA blocks (20 × 12 × 12 mm) were milled through CAD/CAM procedures and used to simulate the alveolar bone. The implant bed was digitally designed and milled. Various diameters of the implant bed were evaluated with the use of Periotest Classic device (Medizintechnik Gulden) so that stability and damping capacity would reflect the mean stability of the implants in human maxilla (−2.0 PTV [53]). The diameter chosen for further manufacturing of experimental models was 3.2 mm. Fifteen 3.5 mm diameter (2.8 mm core diameter) cylindrical dental implants (SPI Element, Thommen Medical AG, Grenchen, Switzerland) were inserted in the blocks with the use of a 3D printed guide to accommodate implant insertion parallel to the long axis of the implant bed. After placing the prosthetic abutments (SPI Easy, Thommen Medical AG), monolithic lithium disilicate (IPS e.max CAD, Ivoclar Vivadent, Schaan, Liechtenstein) single crowns with a flat occlusal surface were milled (*n* = 15) and luted (Single Bond Universal; Relyx Ultimate, 3M ESPE, Maplewood, MN, USA) to the implant-abutment complex with adhesive protocol according to the manufacturer’s instruction (Figure 1).

**Figure 2:**
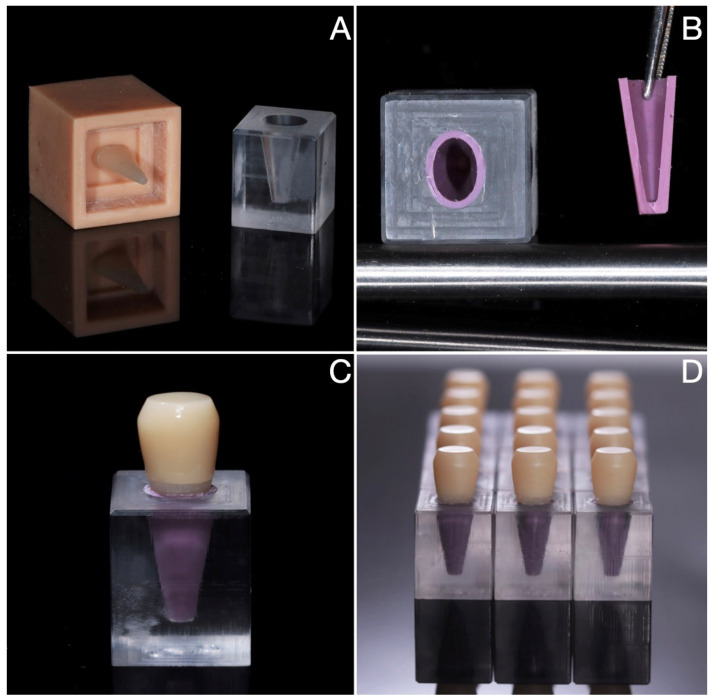
LDS-on-Tooth-Replica samples. Tooth replicas were embedded inside PMMA blocks filled with polyether material using a 3D printed positioner (**A**), which allowed for unambiguous positioning of the replica in the model, leaving an established and homogeneous space around the root (**B**). Monolithic lithium disilicate single crowns with a flat occlusal surface were adhesively luted to each tooth replica (**C**). A total of 15 samples was fabricated (**D**).

For LDS-on-Tooth-Replica, a model of a tooth was designed digitally on the basis of mean dimensions of a second maxillary premolar [54]. The preparation for a full crown with a thickness reduction of 2 mm occlusally and 1.5 mm axially and a total occlusal convergence angle of 8° was designed and milled from a hybrid glass ceramic material (Ambarino High Class, Creamed GmbH & Co., Marburg, Germany) with mechanical properties comparable to human dentin (hardness compared to natural dentin was 710 MPa and 650 MPa, respectively, while modulus of elasticity was 10 GPa and 16.5 Gpa [55,56]). Cubic PMMA blocks with dimensions of 20 × 12 × 12 mm were milled to create a replica of the socket, leaving a space between the tooth root and the socket wall. According to the model adopted in the literature, a polyether material was used as a periodontal ligament imitation [48,50] (Impregum Penta, 3M ESPE). Based on tests using the Periotest Classic device (Medizintechnik Gulden), it was determined that in order to obtain a damping capacity equal to the average characteristics of maxillary teeth (2.5 PTV [53]) the thickness of periodontal space should be 0.85 mm. The polyether adhesive (3M ESPE) was applied on the surface of the roots and the socket. Tooth replicas were embedded inside PMMA blocks filled with polyether material using a 3D-printed positioner, which allowed for unambiguous positioning of the tooth replica in the model, leaving an established and homogeneous space around the root and in the apical area (around 0.85 mm). The positioner also allowed for evacuation of the polyether material excess. After full setting, the excessive polyether material was cut with a scalpel at the level of the top surface of the PMMA cube. Finally, fifteen monolithic lithium disilicate single crowns with a flat occlusal surface were milled (*n* = 15) and adhesively luted (Single Bond Universal; Relyx Ultimate, 3M ESPE) to each tooth replica (Figure 2).

**Figure 3:**
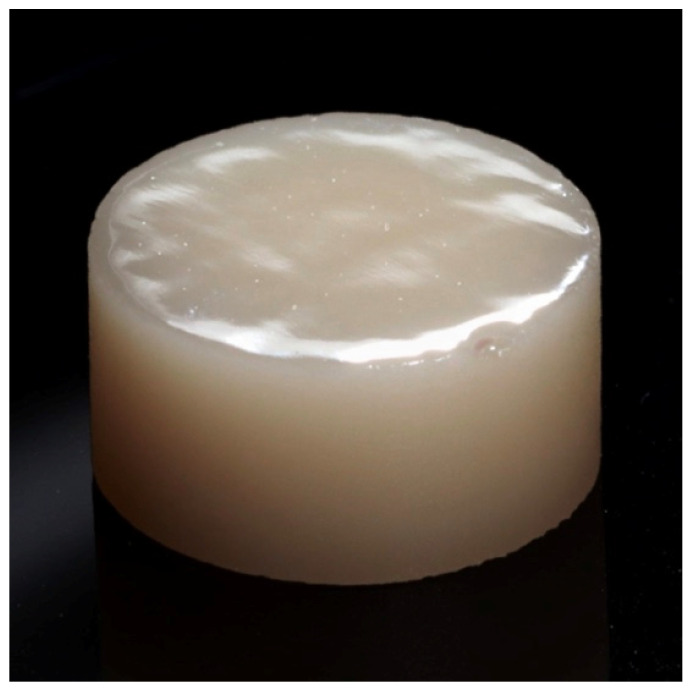
LDS-Cylinders sample.

As for the control group (LDS-Cylinders), 15 cylindrical specimens (*n* = 15) with an 8 mm diameter and a 4 mm height were milled from lithium disilicate blocks with CAD/CAM procedures (Figure 3).

**Figure 4:**
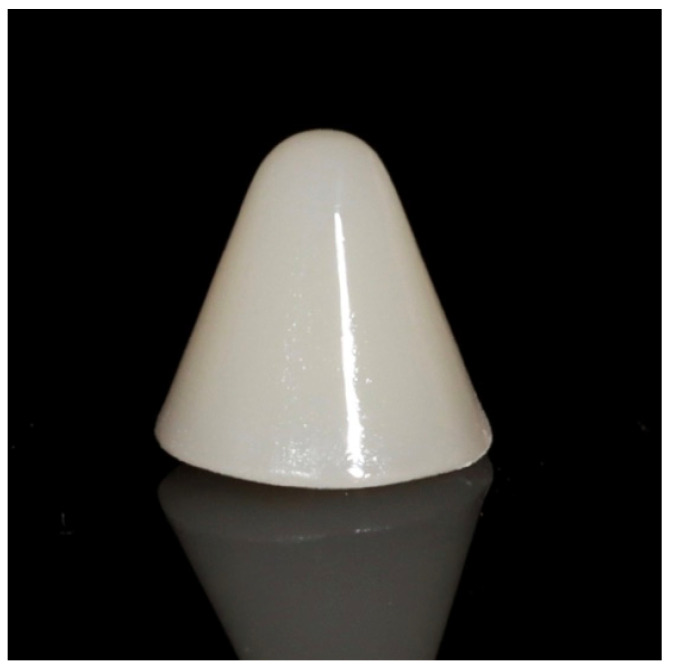
Conical cusp used as antagonist.

Forty-five (*n* = 45) LDS conical cusps with a 2 mm diameter round tip were milled and used as antagonists in all groups (Figure 4) in accordance with the Ivoclar method and several in vitro studies [19,25,26,47,57].

All LDS crowns, LDS cylindrical specimens and LDS antagonistic cusps were carefully polished with medium and fine silicone polishers (Diapro, Eve Ernst Vetter GmbH, Keltern, Germany). No glaze was applied, because, according to the literature, pretreatment of LDS through grinding and polishing demonstrates more favourable wear characteristics than glazing [58,59].

Finally, models from LDS-on-Implant and LDS-on-Tooth-Replica groups were tested with Periotest Classic. Measurements were taken from mesial, distal, buccal and palatal directions and every measurement was repeated 3 times. The mean of 3 measurements was recorded as PT value for a tooth or implant model in measured direction. The mean PT values of 15 models are presented in the Table 1.

### 2.3. Wear Testing

**Figure 5:**
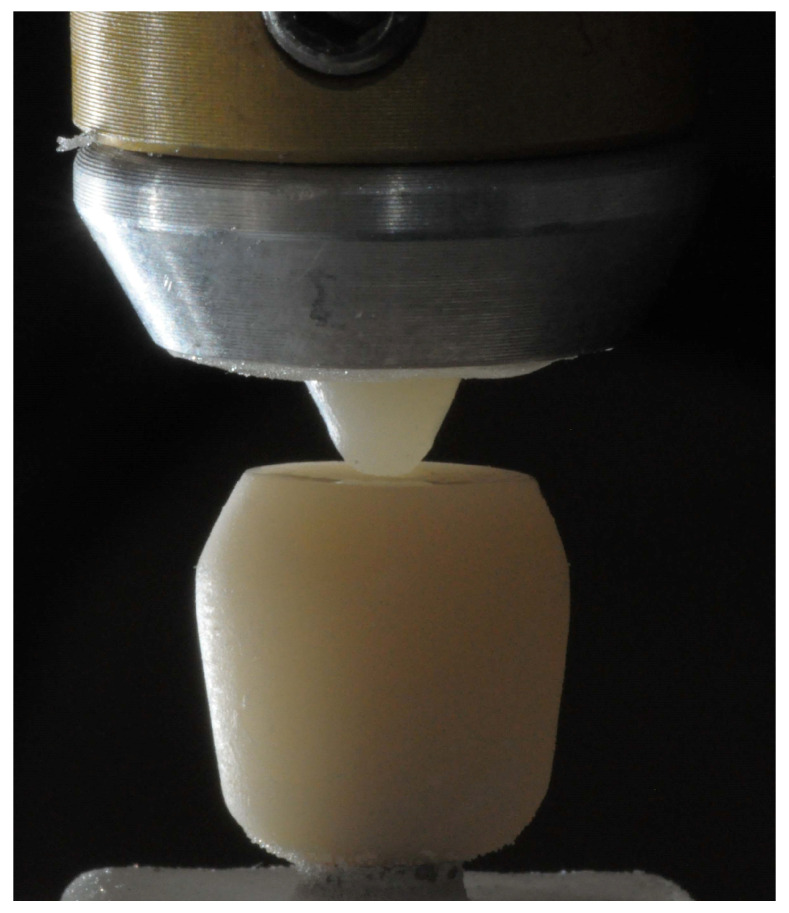
Cusp–sample pair placed in a two-axis chewing simulator.

After being stored at 37 °C for 24 h, each sample of the three experimental groups was randomly paired with an antagonist cusp and placed in a chewing simulator with two axes (CS-4.2, SD Mechatronik GmbH, Feldkirchen-Westerham, Germany) following the methodology described in the literature [19,25,26,47,57]. Acrylic resin (VariDur 200, Buehler, IL, USA) was used to secure the samples inside the chambers and the antagonist cusps in their respective holders (Figure 5).

Subsequently, all sample–cusp pairs underwent a two-body wear test, with parameters as listed in Table 2.

The specimens were subjected to 120,000 cycles while being submerged in artificial saliva throughout the entire test duration.

### 2.4. Data Analysis

After wear test, a quantitative surface assessment was carried out to evaluate the wear facets of the specimens in three dimensions. Three-dimensional meshes of each worn surface were obtained using a laboratory scanner (inEos X5, Dentsply Sirona, Charlotte, NC, USA) in STL (standard triangulation language) format. Subsequently, these meshes were converted to drawing interchange Format (DXF) and imported into computer-aided design software (AutoCAD 2009, Autodesk Inc., San Rafael, CA, USA). The unworn surface surrounding the wear facet was used as reference plane for wear depth (mm) and volume loss (mm^3^) measurement. Cusps were measured both before and after the wear test to assess the detectable linear difference, referred to as antagonist wear (mm). Mean values and standard deviation for wear depth, volume loss and antagonist wear were calculated in each group. SPSS Statistics 24 (IBM Corp., Armonk, NY, USA) statistical software was employed to compare the mean values using three different analysis of variance (ANOVA) tests and the Tukey’s method for multiple comparisons. Normality and homoscedasticity of the data set were respectively confirmed by means of Shapiro–Wilk test and Brown–Forsythe test.

### 2.5. SEM Wear Facet Analysis

After quantitative evaluations, specimens were gold-sputtered and examined under a scanning electron microscope (SEM) (EVO 50 XVP LaB6, Carl Zeiss SMT Ltd., Cambridge, UK). The surfaces were observed at a magnification of 250× to enable the evaluation of the wear facets on the examined specimens. The SEM was operated under the following settings: high vacuum (2107 Torr), a current output of 10 pA, an accelerating voltage of 10 kV, and a working distance of approximately 10 mm.

## 3. Results

**Figure 6:**
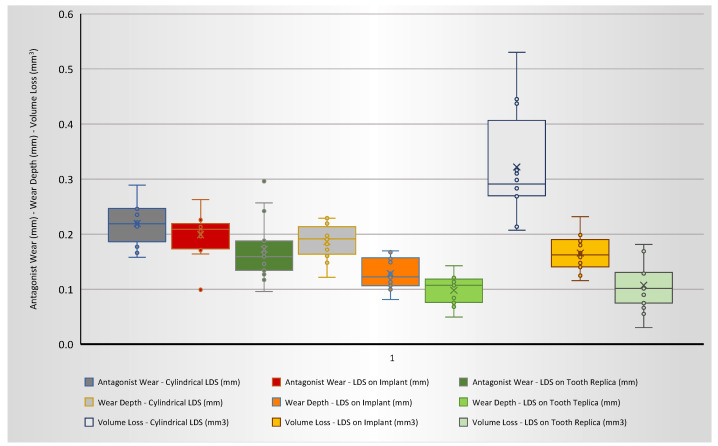
Box plot showing the wear behaviour (in terms of antagonist vertical wear, sample vertical wear depth and sample volumetric loss) of LDS (Lithium Disilicate) cylindrical specimens compared to LDS-implant-supported restorations and to LDS-restorations adhesively luted on tooth replicas.

Mean values and standard deviations recorded in each group for the three dependent variables tested (wear depth, volume loss and antagonist wear) are listed in Table 3 and graphically presented in Figure 6.

The One-Way-ANOVA tests showed that the factor under investigation (abutment rigidity) was able to significantly influence all three variables. In detail, statistically significant differences (*p* < 0.05) were found among the experimental group for both wear depth and volume loss parameters, with the highest values showed by LDS-Cylinders (0.186 mm and 0.322 mm^3^, respectively) and the lowest by LDS-on-Tooth-Replica (0.098 mm and 0.107 mm^3^, respectively). As for antagonist wear, a statistically significant difference (*p* < 0.05) was found between cylindrical LDS (0.220 mm) and LDS-on-Tooth Replica (0.172 mm). The antagonist wear observed on LDS-on-Tooth-Replica was lower than what was observed on LDS-on-Implant (0.199 mm), although no statistically significant differences were detected for this comparison (*p* > 0.05).

**Figure 7:**
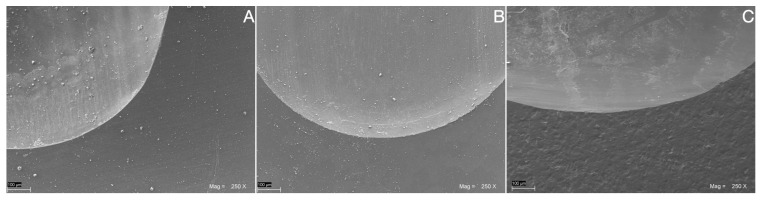
Representative SEM images at 250× magnifications collected for the LDS-on-Implant (**A**), LDS-on-Tooth-Replica (**B**), and LDS-Cylinders (**C**) groups.

Representative SEM images at 250× magnifications were collected for the LDS-on-Implant (Figure 7A), LDS-on-Tooth-Replica (Figure 7B), and LDS-Cylinders (Figure 7C) groups.

A similar wear pattern with smooth scratches running the length of their wear track was evident in all groups. Microcracks were not detectable. Debris was noticeable along the surface, with no difference between worn and unworn areas.

## 4. Discussion

The null hypothesis tested in the present study had to be rejected. The abutment rigidity was a significant factor, able to affect the wear behaviour of LDS restorations. Chewing simulation showed statistically significant differences in both the wear depth and the volume loss variables among LDS-on-Tooth-Replica, LDS-on-Implant and LDS-Cylinders groups. The difference between LDS-on-Tooth-Replica and LDS-on-Implant was 24% in terms of wear depth and 35% in terms of volume loss. This difference indicates that the ability to dissipate the energy of the flexible tooth suspension apparatus may result in a reduced attrition on the prosthetic material itself. The greater energy absorbed by the implant-supported prosthetic restoration results in greater material wear. Data in the literature comparing the wear between implants and natural teeth are lacking, but biomechanical studies confirm that implants may dissipate energy less effectively than natural dentition during loading [22].

A pilot study by Stück et al. evaluating tooth wear through intraoral scans did not show statistically significant differences in wear of the LDS crown on the implant compared to the adjacent natural teeth, nor differences in wear of enamel of antagonistic teeth during the 24-month follow-up period. No comparison between the wear rates of the same prosthetic material supported either by teeth or by implants was performed. This study, as described by the authors themselves, was limited by the small number of cases and the large discrepancy of data related to the multitude of factors affecting tooth wear in the clinical setting [2]. A pilot in vitro study by Rosentritt et al. assessed that the type of abutment (implant or natural tooth) may affect the wear of the same prosthetic material, but the possibility of drawing conclusions from the obtained data was limited [38].

Our study showed significant differences in wear also when implant-supported LDS restorations were compared to cylindrical LDS samples directly embedded within the resin, without any supporting abutment. Wear on LDS-Cylinders was 45% greater in terms of linear depth and 94% greater in terms of volume loss than what was observed on LDS-on-Implant. This indicates some energy dissipation capacity even in the presence of a titanium implant/abutment support, probably also ascribable to the composite cement used for luting the prosthetic restoration [60].

Antagonist wear showed no statistically significant differences between LDS-Cylinders and LDS-on-Implant, and between LDS-on-Implant and LDS-on-Tooth-Replica. Significant differences in antagonist wear between LDS-Cylinders and LDS-on-Tooth-Replica (0.220 mm and 0.172 mm, respectively) were demonstrated. In our study, the effect of the system rigidity on the wear of the antagonistic cusps was less pronounced than that of the test samples. This could be explained, considering that, in all groups, all antagonistic cusps were rigidly fixed to the simulator arm in the same way, without any additional rigid/flexible support.

Two-body-wear, three-body wear, and toothbrushing tests have been described in the literature among the most common in vitro methodologies to investigate wear in ceramic restorative materials [61,62]. The toothbrushing test is used for assessing the abrasion wear of ceramic materials and examining the wear of the glaze or extrinsically stained layers. However, the impact on dental wear can vary significantly depending on various factors, such as the technique and timing of toothbrushing, the type of dentifrice and toothbrush employed, as well as the frequency and force of brushing [62]. The three-body wear test replicates clinical conditions to simulate occlusal wear through the introduction of a third abrasive body between the ceramic surface and the opposing surface simulating food bolus. Studies on composite and amalgam samples revealed that gradual change in the distance between the opposing substrates and even minor alterations of the abrasive-film features and thickness at the contact areas result in significant changes in wear rates and wear-rate ranking of the materials [61,62]. Two-body wear tests are suitable for predicting the wear behavior of dental materials [44] through the simulation of non-masticatory tooth movements such as swallowing, empty mastication, parafunctions and dynamic occlusion movements [63]. Moreover, a two-body wear test using standardized antagonists made out of the same material of the tested specimens allows simulation of the situation of restored occlusal surfaces occluding in a full-mouth rehabilitation following the treatment of a severely worn patient [20,26]. In the present paper, two-body wear test of LDS samples resulted in a SEM surface topography characterized by smooth wear tracks surrounded by debris. In dental ceramics, wear is strongly affected by surface roughness [64,65,66]. The repeated sliding contact on the ceramic surface leads to compressive stresses before movement, shear stresses at the contact interfaces, and tensile stresses at the trailing edge of the antagonist, thus resulting in fatigue wear and debris formation able to further increase wear [61,67]. Despite some controversies [68], several studies demonstrated greater wear for materials opposed to glazed ceramics than to polished ones [68,69,70]. Polished LDS showed a tribological behaviour close to human enamel [59,69] and should therefore be preferred to glazed LDS for single restorations [58].

According to Brosh et al., elastomeric dental impression materials perform similarly to periodontal ligament under loading and also in terms of immediate recovery when the load is not applied and thus are the material of choice in PDL simulations [71]. The presented methodology of making teeth replicas with periodontium using CAD/CAM technology showed high repeatability. The standard deviation of the measurements of damping capacity using the Periotest was 0.91 (on a scale of −8 to 50). Obtained Periotest values were comparable with those described for natural teeth and implants, and the appropriate difference in vibration damping between teeth replicas and implants was achieved [53]. The homogeneity of the obtained models made it possible to control the variables that could affect the tested parameters. The width of the periodontal gap used in the present model was around 0.85 mm, similar to a previously described in vitro model in which tooth mobility was directly measured to reflect mobility of natural dentition (0.075 ± 0.1 mm) [50]. These data indicate that simulating periodontal ligament with polyether material should take into account a larger periodontal gap width than that observed in natural tissues (0.15–0.38 mm) [72] to obtain adequate mobility and damping capacity of the in vitro models. The presented methodology made it possible to eliminate the previously described problem of obtaining a reproducible thickness of the silicone layer simulating PDL by manually soaking the root in wax [51,52], as well as reduced the time needed for model fabrication [50].

A limitation of the present approach was the use of solid PMMA blocks, which, according to the technical data, have a similar modulus of elasticity as human bone (approx. 3000 MPa) [73,74]. However, reproducing the trabecular and cortical bone layer with a material that would precisely emulate the properties of human bone might bring the presented model closer to the clinical situation.

In the present study, tooth replicas milled from hybrid ceramics with a similar modulus of elasticity and compression resistance as human dentine [75] were used instead of natural teeth. Despite the fact that the use of natural tissues would be the closest to clinical conditions, natural teeth are characterized by large discrepancies in shape, but also in mechanical properties, which results in increased inhomogeneity of the results [38]. The described experimental model is easy to reproduce and relatively time effective to perform, which allows for a wider application in the in vitro study of prosthetic materials.

## 5. Conclusions

Considering the limitations of the presented experimental model, it can be concluded that:

1. The rigidity of the abutment is able to significantly affect the wear pattern of lithium disilicate glass ceramics, which seems to be more intense in the presence of a rigid implant-abutment support than on restorations supported by more flexible teeth replicas and in the presence of periodontal ligament.
2. In vitro chewing simulation models that include a wider number of intra-oral parameters (alveolar bone, periodontal ligament, abutment rigidity/elasticity, etc.) might be closer to the actual clinical situation and seem to produce significantly different results from extremely simplified in vitro models.

## Figures and Tables

**Table 1:** Mean Periotest values of experimental models (on the scale from −8 to 50).

	LDS-on-Tooth-Replica		LDS-on-Implant	
Direction	Mean (PTV)	SD	Mean (PTV)	SD
Buccal	2.88	1.13	−1.38	0.84
Palatal	2.91	1.04	−1.71	0.88
Mesial	3.04	0.73	−1.69	0.51
Distal	3.14	0.75	−1.97	0.57
Overall	2.99	0.91	−1.69	0.73

**Table 2:** Chewing simulator setting parameters.

Number of Cycles	120,000
Force	49 N
Height	3 mm
Lateral movement	−0.7 mm
Lowering speed	60 mm/s
Lifting speed	60 mm/s
Advanced speed	40 mm/s
Return speed	40 mm/s
Frequency	1.6 Hz

**Table 3:** Mean values (and standard deviations) recorded for antagonist vertical wear (mm), sample vertical wear depth (mm) and sample volumetric loss (mm^3^) within the three experimental groups under investigation.

	Antagonist Wear (mm)	Wear Depth (mm)	Volume Loss (mm^3^)
LDS-Cylinders	0.220 ^a^	0.186 ^a^	0.322 ^a^
	(0.038)	(0.032)	(0.098)
LDS-on-Implant	0.199 ^a,b^	0.128 ^b^	0.166 ^b^
	(0.038)	(0.028)	(0.033)
LDS-on-Tooth-Replica	0.172 ^b^	0.098 ^c^	0.107 ^c^
	(0.055)	(0.026)	(0.045)

Same superscript letters indicate not statistically significant differences (reading vertically).

## Data Availability

The data presented in this study are available on request from the corresponding author.
